# Polarity Specific Effects of Cross-Hemispheric tDCS Coupled With Approach-Avoidance Training on Chocolate Craving

**DOI:** 10.3389/fphar.2018.01500

**Published:** 2019-01-24

**Authors:** Sandra Carvalho, Adriana Sampaio, Augusto J. Mendes, Alberto Lema, Daniela Vieira, Óscar F. Gonçalves, Jorge Leite

**Affiliations:** ^1^Neurotherapeutics and Experimental Psychopatology Group, Psychological Neuroscience Laboratory, Centro de Investigação em Psicologia (CIPsi), School of Psychology, University of Minho, Braga, Portugal; ^2^Department of Physical Medicine and Rehabilitation, Spaulding Neuromodulation Center, Spaulding Rehabilitation Hospital, Harvard Medical School, Boston, MA, United States; ^3^Portucalense Institute for Human Development (INPP), Universidade Portucalense, Porto, Portugal

**Keywords:** chocolate craving, approach-avoidance training, tDCS, PFC, implicit preference

## Abstract

Transcranial Direct Current Stimulation (tDCS) over the Dorsolateral Prefrontal Cortex (DLPFC) has already been shown to decrease craving for food. However, it remains unclear whether a single session of tDCS combined with a cognitive bias modification (CBM) task may affect explicit and implicit measures of craving for chocolate. Fifty-one healthy volunteers (38 females; mean age: 22.12 ± 3.38) were randomly allocated to CBM training based on the Approach Avoidance task and either Sham, Right anodal-Left cathodal (RALC), or Left anodal-Right cathodal (LARC) tDCS. Results show that there was an increase in the explicit craving for chocolate, as assessed by the Visual Analog Scale [*F*(2, 46) = 3.239, *p* = 0.048], from the baseline to post-intervention. Participants which received LARC tDCS were explicitly self-reporting more craving for chocolate than those that received RALC tDCS (*p* = 0.023). Moreover, this effect was also observed on the implicit measure [*F*(2, 46) = 4.168, *p* = 0.022]. LARC tDCS significantly increased the implicit preference for chocolate when comparing to both RALC (*p* = 0.009) and Sham tDCS (*p* = 0.034). Previous studies have shown that RALC tDCS over the PFC is able to effectively decrease craving for food. Interestingly, the present data not only does not reproduce such result, but instead it suggests that LARC tDCS can actually increase the preference for chocolate. This result is compatible with recent models of brain laterality, in which cue craving seems to be more dependent on the left hemisphere. Thus, shifting the activity to the left hemisphere (while simultaneously reducing the activity over the homotopic region) may have led to this increased implicit as well as explicit preference for chocolate.

## Introduction

Food craving refers to an intense motivational state (i.e., desire) to eat high caloric food (Avena et al., 2009; Hormes and Rozin, 2010). Depending on the pattern of severity, intensity, and frequency, food craving can become a significant burden, especially if associated to binge-eating episodes, which can significantly influence individual’s health, namely by developing diabetes. and/or cardiovascular disorders (Polivy et al., 2005). In addition, food craving is thought to be extremely relevant for individuals suffering from obesity and/or eating disorders (Potenza and Grilo, 2014). For instance, craving for food, prior to exposure, has been associated with increased food intake in people suffering from binge eating disorders or obesity (Ng and Davis, 2013). According to the World Health Organization in 2012, overweight and obesity are the cause of 2.8 million deaths every year, and thus better pharmacological and non-pharmacological interventions to decrease food craving are required.

From a cognitive standpoint, it is thought that craving for unhealthy and addictive food is reinforced by an implicit cognitive biased process (cognitive bias) that results from being exposed to environmental cues that target desire for that specific type of food (Brockmeyer et al., 2015). These cognitive bias modification (CBM) interventions for attention and approach to food have been used recently with some success (see Fodor et al., 2017 for meta-analysis).

Another intervention that has yield promising results in terms of reducing food craving is transcranial direct current stimulation (tDCS). Several studies have shown that in the presence of high caloric food, there is greater bilateral activation in the medial prefrontal cortex (mPFC), dorsolateral prefrontal cortex (DLPFC), thalamic regions, among others (Killgore et al., 2003). There is also evidence that tDCS over the PFC is able to enhance inhibitory control while reducing food craving and food intake (Kekic et al., 2014; Lapenta et al., 2014; Georgii et al., 2017), as well as the desire for specific foods (Fregni et al., 2008). In a randomized, sham –controlled, cross-over study by Fregni et al. (2008) participants received bilateral tDCS (anode left/cathode right and anode right/cathode left) to the DLPFC and sham tDCS. Results show that participants reported increased craving and food intake after sham tDCS, which was not observed after active bilateral tDCS (anode left/cathode right). Additionally, participants consumed less food after both active tDCS conditions and engaged less pictures of food after receiving anode right/cathode left tDCS, as assessed by eye-tracking. Another study (Lapenta et al., 2014) using an anode right/cathode left tDCS montage showed that this reduction in caloric intake was associated with frontal N2 ERP component reduction and enhanced P3a ERP component in response to No-go stimuli, which have been interpreted as being markers of inhibitory control.

However, none of these studies explored the synergetic effect of combining tDCS to the PFC with CBM designed to train avoidance to chocolate stimuli. Chocolate is one of the most craved foods in western countries (Meule and Hormes, 2015; Richard et al., 2017), because its high energy density and its association with pleasant emotions (Pandolfi et al., 2016). Moreover, past studies have not combined CBM with tDCS in order to maximize the effects on food craving reduction of each other, nor have been using implicit measures for assessing the effects of such interventions on food craving. The use of implicit measures is important, especially because explicit measures are prone to social desirability bias (Krieger et al., 2011; Echabe, 2013).

Thus, the main objective of this work was to test if active bilateral tDCS combined with a modified approach avoidance task for chocolate (AAT) is able to reduce chocolate craving on implicit (i.e., implicit association task – IAT) and explicit measures (visual analog scale – VAS) when comparing to another high caloric food, namely fast food. The hypothesis underlying the objective of this study is that the synergetic effect between the active tDCS combined with a modified version of AAT to train avoidance to chocolate stimuli, will decrease chocolate preference as compared to cognitive training alone (i.e., combined with sham tDCS).

## Materials and Methods

### Participants

A total of 51-college student volunteers (38 females; mean age: 22.12 ± 3.38) naïve to tDCS and CBM participated in this study. Kekic et al. (2014) showed that active tDCS was able to significantly reduce craving for sweets (*M* = 13.31, *SD* = 8.44) when comparing to sham (*M* = 6.06, *SD* = 8.66). For our study we increased the expected effect size in 20% (in order to be conservative as we are testing the combination of CBM with tDCS and as such the effect of the combined interventions should be superior to tDCS alone) and thus with a Cohen’s d of 1.02, power of 0.80 and with two sided alpha set at 0.05, we would need to enroll at least 17 participants per group.

All participants were right-handed (Edinburgh Handedness Inventory: EHI ≥ 80), healthy, with normal or corrected-to-normal visual acuity and without present or past history of neurological or psychiatric disorders. Participants were excluded if they were using any medication or psychotropic drugs at the time of the study, if their body mass index (BMI) was below 18.5 or above 25; or if they had clinically relevant levels of anxiety or depression as assessed by the State and Trait Anxiety Inventory (STAI-Y; Spielberger and Gorsuch, 1983) and the Beck Depression Inventory (BDI-II; Beck et al., 1996) (Table 1). Participants were advised to avoid alcohol, cigarettes and caffeinated drinks on the day of the experiment, and none reported fatigue due to insufficient sleep. The study was performed in accordance with the Declaration of Helsinki an all participants gave their written informed consent prior to their inclusion in the study. The study was approved by the local ethics committee – *Subcomissão de Ética para as Ciências da Vida e da Saúde* (SECVS) – SECVS 010/2016.

**Table 1:** Sociodemographic information.

		Stimulation		
	Total (*N* = 51)	Sham (*N* = 17)	RALC (*N* = 17)	LARC (*N* = 17)
**Sex**				
Male	13	4	5	4
Female	38	13	12	13

	**Mean (*SD*)**	**Mean (*SD*)**	**Mean (*SD*)**	**Mean (*SD*)**

Age	22.12 (3.38)	22.65 (5.05)	22.41 (2.55)	21.29 (1.61)
BMI	22.04 (2.79)	21.75 (2.18)	22.88 (3.03)	21.49 (3.04)
BDI	4.67 (4.54)	4.24 (4.75)	5.35 (4.77)	4.41 (4.27)
**STAI-Y**				
State	28.14 (6.70)	26.65 (4.27)	29.06 (7.95)	28.71 (7.44)
Trait	32.37 (8.43)	33.24 (9.18)	31.06 (7.81)	32.82 (8.58)


*RALC, right anodal-left cathodal; LARC, left anodal-right cathodal; BMI, body mass index; BDI, Beck Depression Inventory; STAI-Y, State-Trait Anxiety Inventory*.

### Design

#### Overall Procedure

This experiment consisted of assessing implicit and explicit preference for chocolate before and after active or sham tDCS combined with CBM [using a modified version of the Approach Avoidance Task (AAT)]. In the pre- and post-task assessments participants were screened about possible levels of discomfort, fatigue, pain, itching, humor, tingling, burning, headache and sleepiness (among others) using a continuous Visual Analog Scale (VAS). In this scale with interval-level measurements, participants could choose any value from 0 (“absent”) to 10 (“Maximum of”).

**FIGURE 1:**
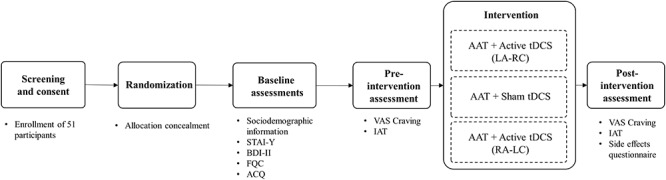
Schematic representation of the study. AAT, Approach Avoidance Task; tDCS, transcranial Direct Current Stimulation.

The objective of this assessment was to evaluate possible secondary effects due to the stimulation. Then participants performed the Implicit Association task (IAT). Following these baseline assessments, participants then received online active or sham tDCS while they were performing the CBM training (i.e., Approach Avoidance Task). Participants received tDCS for a total of 20 min. tDCS started 3 min before CBM training and remained on during the entire duration of the training (participants took approximately 15 min to complete the CBM training). After tDCS, participants performed the post-intervention assessment which included the IAT (Figure 1).

#### Intervention

##### Cognitive training task: approach- avoidance task (AAT)

**FIGURE 2:**
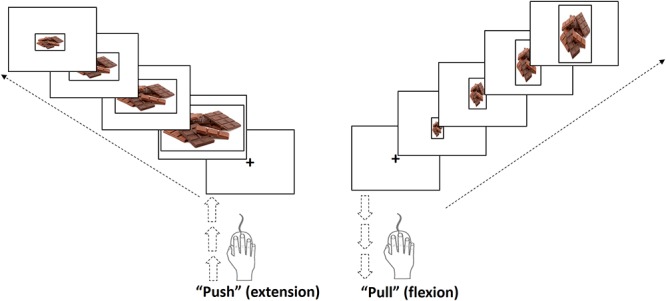
Cognitive training task: a modified version of the approach- avoidance task (AAT).

The AAT measures the approach and avoidance bias toward specific categories (Rinck and Becker, 2007). However, this task can be modified to train individuals to avoid or approach specific targets, namely by what has been called CBM. During CBM training, participants were trained to avoid more often chocolate pictures than fast food, healthy food, and neutral (objects) pictures. During this task (adapted from Wiers et al., 2009) participants were comfortably seated in front of a computer. Participants had to “pull” or “push” the computer mouse according to the portrait or landscape format of the images presented on the computer screen. There were 400 trials in total (100 per category). For the CBM procedure for chocolate, the probability was set at 80% for push (i.e., avoid) and 20% for pull (i.e., approach), while for the remaining categories the push/pull probability was set at 50% (Figure 2).

##### Transcranial direct current stimulation (tDCS)

2 mA intensity tDCS was applied for 20 min using 35 cm^2^ saline-soaked electrode sponges, through an Eldith DC Stimulator Plus (Neuroconn, Germany). The experiment had a three arm parallel design, in which participants were randomized to receive one of 3 tDCS conditions: anode right/cathode left (RALC); cathode right/ anode left (LARC) and sham tDCS. Electrodes were placed over F3 and F4 according to the 10–20 system with at least 6 cm distance between them, following previous studies (Leite et al., 2013; Kekic et al., 2014, 2017). DC was applied, during the entire duration of the task (with a 15 s ramp up and down) for the active condition, while for the sham condition the duration was 15 s (with a 15 s ramp up and down).

#### Assessments

##### Implicit association task (IAT)

Chocolate implicit or automatic preference was assessed by the IAT before and after the intervention (i.e., AAT coupled with tDCS). This task measures implicit preference for specific categories, by assessing strength of association between specific word concepts (for instance, good and bad) and images representing a specific category (for instance, food). We adapted this task to assess the implicit preference for specific foods, such as chocolate (chocolate cake, chocolate bar), healthy snacks (such as fruit and vegetables), fast food (hamburgers, pizza), and neutral images (objects) (see Supplementary Material for Task performance on the multifactorial IAT).

This task was based on the brief IAT procedure proposed by (Sriram and Greenwald, 2009) in which two contrasted contents are presented on the computer screen in the format of words and images. On top of the screen categories are displayed (i.e., Chocolate pictures and “Good” Words) and then target word and picture stimuli (one at a time) are displayed on the center of the screen. Subjects had to respond as fast as they could by pressing one of two keys: “I” for the target words and pictures categories and “E” for all other words and pictures. “I” and “E” keys indicate a right and left hand response, respectively. For instance, if the target word category displayed on screen was “Good” and the picture category was “Chocolate” participants should respond “I” if the word presented on screen was from the “good” category, or if the picture depicted was from the chocolate category. They should respond “E” for any other words or pictures content.

The “bad words used in this task were: unpleasant, disgusting, nasty, and tasteless; while delicious, tasty, pleasurable, and pleasant were used as words for the “good” category. Throughout the task, the association between the word categories and picture content varied from bock to block. Every time the participant made a mistake, a small red “X” was presented in the center of the computer screen indicating a wrong answer, and thus participant should correct the response before proceeding to the next trial. In total there were 12 blocks (comparing all the combinations between picture and word categories) with a total of 240 trials. Responses were self-paced, and there was a 250 ms inter-trial interval. D scores were calculated using the improved algorithm suggested by Greenwald et al. (2003), in which the standardized mean difference of latencies between hypothesis consistent (i.e., letter and picture categories) and hypothesis inconsistent pairings (i.e., letter and picture categories) was assessed. In order to preprocess the data, trials over 10,000 ms were excluded, as well as participants’ removal if more than 10% of the trials have a latency bellow 300 ms (no participants were removed). Then correct trials are averaged and their standard deviations pooled. The mean difference was then divided by the target trials pooled SD.

##### Explicit preference for chocolate

Explicit measure of chocolate craving: participants rated their chocolate craving before and after the intervention by answering to the question “If you had chocolate available at this moment, You …” on a VAS ranging from 1 to 10 (1 = “Would not eat any chocolate” and 10 = “Would certainly eat chocolate”).

##### Attitudes to Chocolate Questionnaire – ACQ

The Attitudes to Chocolate Questionnaire (ACQ) is a 24-item self-report instrument that assesses attitudes toward chocolate such as craving (10-itens), guilt (10-itens), and functional approach (4-itens) (Benton et al., 1998).

##### State and Trait Food Cravings Questionnaire – FCQ-S and FCQ-T

The State and Trait Food Cravings Questionnaire is a self-report questionnaire to assess state and trait craving for chocolate and other foods (Cepeda-Benito et al., 2000).

The Food Cravings Questionnaires-Trait reduced (FCQ-T-r) assesses: (1) lack of control over eating, (2) thoughts or concerns with food, (3) plans and intentions for food consumption, (4) emotions before or during food consumption, and (5) environment or cues that elicit craving for food. For the state version, *Food Cravings Questionnaire-State* (FCQ-S) comprehends 15 items assessing general food craving and 15 items to assess chocolate craving (Table 2).

**Table 2:** Measures of craving and consumption of food and chocolate.

		Stimulation				
Total		Sham	RA-LC	LA-RC		
(*N* = 51)		(*N* = 17)	(*N* = 17)	(*N* = 17)	*F*(2, 48)	*P*
Mean (*SD*)		Mean (*SD*)	Mean (*SD*)	Mean (*SD*)		
**Food-craving questionnaire – trait general craving**						
**General craving**						
Lack of control	2.04 (0.9)	1.99 (0.9)	1.95 (0.92)	2.18 (0.9)	0.29	0.75
Thoughts	1.96 (0.78)	1.75 (0.57)	1.87 (0.73)	2.27 (0.94)	2.14	0.13
Plans	2.63 (1.01)	2.65 (0.7)	2.26 (1.05)	2.97 (1.17)	2.16	0.13
Emotions	2.78 (1.08)	3.06 (0.79)	2.47 (1.04)	2.82 (1.33)	1.29	0.29
Environment	3.10 (1.22)	3.24 (0.9)	2.94 (1.39)	3.12 (1.36)	0.24	0.79
Total	2.26 (0.7)	2.24 (0.53)	2.1 (0.7)	2.46 (0.84)	1.13	0.33
**Chocolate craving**						
Lack of control	2.19 (0.83)	2.15 (0.86)	2.22 (0.97)	2.19 (0.70)	0.03	0.97
Thoughts	1.87 (0.64)	1.73 (0.54)	2.00 (0.74)	1.89 (0.64)	0.76	0.48
Plans	2.40 (0.96)	2.41 (0.73)	2.26 (1.13)	2.53 (1.01)	0.32	0.73
Emotions	2.82 (1.10)	3.09 (0.83)	2.59 (1.18)	2.79 (1.25)	0.88	0.42
Environment	3.04 (1.26)	3.06 (1.20)	3.12 (1.58)	2.94 (1.03)	0.08	0.92
Total	2.25 (0.63)	2.23 (0.53)	2.26 (0.78)	2.27 (0.58)	0.01	0.98
**Attitudes to chocolate questionnaire – ACQ (cm)**						
Craving	2.70 (1.63)	3.10 (1.85)	2.60 (1.74)	2.50 (1.29)	0.69	0.51
Guilt	1.10 (1.60)	1.10 (1.91)	1.20 (1.39)	1.10 (1.56)	0.09	0.91
Functional approach	2.80 (1.60)	2.80 (1.66)	2.50 (1.28)	3.70 (1.67)	2.77	0.07
Total	2.30 (1.60)	2.80 (1.66)	2.10 (1.28)	2.40 (1.67)	0.45	0.63


*RALC, right anodal-left cathodal; LARC, left anodal-right cathodal.*

##### Side effects questionnaire

tDCS side effects questionnaire: participants completed this questionnaire after the tDCS session to evaluate potential adverse effects of tDCS, such as tiredness, anxiety, sadness, agitation, sleepiness, itching, headache, pain, tingling, and metallic taste in the mouth) in a continuous VAS scale, ranging from 0 to 10 (0 = absent and 10 = maximum of). Blinding to the tDCS condition was also assessed, by asking the participants at the end of the session “Which type of tDCS did you received?” Participants could respond between “Active,” “Sham” or “I do not know.”

### Statistical Analysis

Two participants were not included in the analysis because they did not complete all the assessments. Comparisons between groups (RALC, sham, LARC) regarding attitudes toward food and chocolate craving (i.e., FCQ and ACQ) were assessed with using one-way independent samples ANOVA at baseline. Scores were calculated as a delta, namely the difference between the scores after intervention and the ones on the baseline. We carried out two independent ANOVAs, after using exploratory data analysis. If homogeneity of variances was not assumed, we also conducted the Brown-Forsythe test. It is important to note that the main objective of the present study was to compare the effects of the combination of CBM and tDCS in craving specific for chocolate, when comparing to other high caloric foods, such as fast food. Therefore, in order to test the primary outcome, namely the implicit preference for chocolate when comparing to fast food, a one-way independent samples ANOVA was performed with three levels (RALC, sham, LARC). Another independent samples ANOVA was performed for the explicit craving for chocolate, as measured by the VAS. The statistical significance was set at 0.05, and if the main effect of the ANOVA was significant, *post hoc* Fisher’s LSD tests were used. Additionally, in order to categorize decrease or increase in craving after stimulation, Fischer’s exact test was used. All data analyses were performed using SPSS version 21 (IBM, United States).

### Data Availability

Raw data is freely available to any scientist without breaching participant confidentiality and for non-commercial purposes. In order to have access to the raw data please contact the corresponding author.

## Results

There were no serious or adverse side effects reported due to the intervention (please see Table 3). The most reported side effects were tiredness and sleepiness (see Table 3). RALC significantly increased the anxiety levels when compared to sham, however, the average anxiety for RALC was of 1.38 (2.13) on a 10-point scale. Moreover, only 22 participants (44.90%) were able to correctly guess whether they were on active or sham tDCS.

**Table 3:** Side effects after intervention – visual Analog scale [from 0 (absent) to 10 (maximum of)].

	Sham	LARC	RALC
	Mean (*SD*)	Mean (*SD*)	Mean (*SD*)	*F*
Tiredness	3.50 (1.60)	3.38 (3.20	4.88 (2.59)	n.s
Anxiety	0.13 (0.35)	0.50 (0.93)	1.38 (2.13)	0.03 ^∗^
Sadness	0	0.25 (0.71)	0.25 (0.46)	n.s.
Agitation	0.38 (0.74)	0.25 (0.71)	0.50 (0.53)	n.s.
Sleepiness	2.38 (1.41)	3.38 (3.16)	3.13 (2.47)	n.s.
Itching	1 (1.41)	2.50 (2.27)	2.50 (2.27)	n.s.
Headache	0.75 (0.89)	0.38 (0.52)	0.50 (0.53)	n.s.
Pain	0.13 (0.35)	0	0	n.s.
Tingling	0.50 (0.76)	1.50 (1.93)	0.50 (1.07)	n.s.
Metallic taste	0	0.63 (1.41)	0	n.s.


*RALC, right anodal-left cathodal; LARC, left anodal-right cathodal ^∗^ significant difference between RALC and sham.*

Exploratory data analysis showed that skewness was 0.039 and 1.047, while Kurtosis was 0.023 and 0.870 for the implicit and the explicit measure, respectively.

### Implicit Measures of Chocolate Craving

**FIGURE 3:**
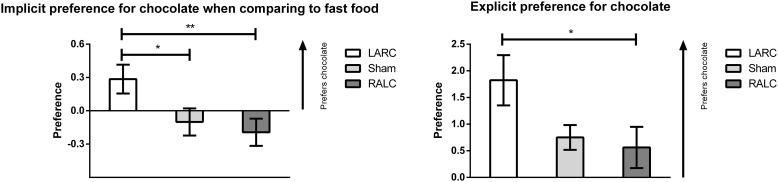
Implicit and explicit change from baseline in terms of preference for chocolate (^∗^*p* < 0.05; ^∗∗^*p* < 0.01).

In order to test the primary outcome, we calculated the delta between the d’scores before and after stimulation. A positive delta score means an increase in chocolate preference. The main analysis showed that there was a main effect for the implicit preference after stimulation for chocolate when comparing to fast food [*F*(2, 46) = 4.168, *p* = 0.022]. *Post hoc* comparisons showed that those that were submitted to the LARC condition (*M* = 0.285, *SE* = 0.130) significantly increased their preference for chocolate when comparing to both RALC (*M* = -0.193, *SE* = 0.123) (*p* = 0.034) and sham (*M* = -0.101, *SE* = 0.122) (*p* = 0.009). There were no main effects of tDCS on the implicit preference between chocolate and healthy food [*F*(2, 46) = 0.275, *p* = 0.761], nor between healthy and fast food [*F*(2, 46) = 1.055, *p* = 0.357] (see Figure 3 and Table 4).

**Table 4:** Chocolate preference.

	Stimulation		
	Sham Mean *(SD)*	RALC Mean *(SD)*	LARC Mean *(SD)*
**Versus fast food – IAT**			
Pre	0.25 (0.50)	0.46 (0.45)	0.02 (0.55)
			
Post	0.15 (0.40)	0.27 (0.42)	0.31 (0.40)
			
**Explicit preference – VAS**			
Pre	5.50 (3.01)	4.31 (3.17)	3.82 (1.94)
Post	6.25 (3.13)	5.00 (3.54)	5.52 (2.55)


*d scores for chocolate implicit preference when comparing to fast food (higher scores mean higher preference for chocolate) and explicit preference scores. There were no significant differences in terms of implicit and explicit preference for chocolate in terms of baseline between the groups (p > 0.05). RALC, right anodal-left cathodal; LARC, left anodal-right cathodal; IAT, Implicit Association Task; VAS, Visual Analog Scale.*

We conducted a further analysis exploring if there were differences in terms of craving reduction for chocolate, by analyzing the reduction in terms of preference for chocolate. Table 5 summarizes the data:

**Table 5:** Number of participants in which the craving was reduced following the intervention.

	Decrease in craving	
Condition	No	Yes
LARC + CBM	11	6
Sham + CBM	5	11
RALC + CBM	4	12


*RALC, right anodal-left cathodal; LARC, left anodal-right cathodal; CBM, cognitive bias modification.*

Fisher’s exact test suggested a significant difference between stimulation conditions [χ^2^(2) = 6.001, *p* = 0.048] in terms of craving reduction. There were no significant differences between Sham and RALC in terms of craving reduction conditions [χ^2^(1) = 0.155, *p* = 0.694], nor between Sham and LARC [χ^2^(1) = 3.694, *p* = 0.055]. But there was a significant difference between LARC and RALC [χ^2^(1) = 5.241, *p* = 0.020].

### Explicit Measures of Chocolate Craving

Interestingly enough this implicit preference was accompanied by changes in the explicit preference for chocolate as assessed by the VAS [*F*(2, 46) = 3.239, *p* = 0.048]. The Brown-Forsythe test also supported this claim [*F*(2, 37.72) = 3.303, *p* = 0.048]. *Post hoc* comparisons showed that LARC (*M* = 1.824, *SE* = 0.472) significantly increased the preference for chocolate when comparing to RALC (*M* = 0.563, *SE* = 0.387) (*p* = 0.023) but not sham (*M* = 0.750, *SE* = 0.233) (*p* = 0.052).

## Discussion

In the present study, the combination of CBM and active tDCS was not able to reduce craving for chocolate. Moreover, it seems that combining left anode and right cathode tDCS with CBM actually increases chocolate craving. Previous research has shown that anode to the right and cathode to the left dorsolateral pre-frontal cortex (DLPFC) was able to reduce food craving; and that left anode right cathode did not increase food craving when compared to sham (Fregni et al., 2008). Thus, the results of the present study seem to be contradictory to what has been reported previously in the literature (Kekic et al., 2014; Shahbabaie et al., 2014; den Uyl et al., 2017; Hall and Lowe, 2018; Lapenta et al., 2018).

It is important to highlight that previous studies did not combine tDCS with CBM in order to reduce craving. In fact, most of the previous studies have been using tDCS alone and assessing its potential effects on craving (Coles et al., 2018; Mostafavi et al., 2018). Moreover, when tDCS seems to be combined with a task, state dependent effects have been reported. For instance, Shahbabaie et al. (2014), showed that anode to the right and cathode to the left PFC was able to reduce craving in methamphetamine users at rest, however, craving increased during a computerized cue-induced craving task. This study clearly suggests that in the absence or presence of craving inducing cues, there is a shift in the direction of the tDCS-induced effects in a state dependent manner that goes beyond the neurophysiological effects of anodal and cathodal stimulation (Nitsche and Paulus, 2000; Nitsche et al., 2003). These state dependent effects rely on the interaction between tDCS and several other factors, such as baseline level of activation (Fregni et al., 2006; Carvalho et al., 2015), dose (Zaghi et al., 2010), task and timing specific effects (Pirulli et al., 2013; Saucedo Marquez et al., 2013; Bortoletto et al., 2015) or broader network activation. In fact, 20 min of 2 mA tDCS has already been shown to increase the activity in the anticorrelated network (AN) which is thought to be activated during focused attention (Peña-Gómez et al., 2012). Thus suggesting that the effects of tDCS are not limited to the stimulated brain region, but in fact are able to modulate large-scale brain networks.

Moreover, there may be a laterality effect in terms of risk factors for addiction (Gordon, 2016). Impulsivity seems to be more related to the right hemisphere, while cue induced craving seems to be more associated to the left hemisphere, as shown by BOLD changes. It is plausible that the anode over the right hemisphere and the cathode over the left may actually decrease craving, especially in the absence of craving inducing cues, whereas the anode to the left hemisphere could result in an overall net excitement of increasing craving during cue exposure. tDCS to the right PFC has already been shown to increase inhibitory control (e.g., Leite et al., 2018), and as such it is possible that the anode on the right hemisphere led to a net excitement that by increasing inhibitory control/decreasing impulsivity is able to decrease craving. However, the anode on the left PFC on a region that is activated during cue exposure let to a net excitement that actually increased craving.

In fact, in the present study, anode to the right and cathode to the left PFC combined with CBM decreased the preference for chocolate, when comparing to fast food, which is in line with previous studies (Brockmeyer et al., 2015). This trend for decrease was also observed for sham in combination with CBM (*p* = 0.055) when comparing to the LARC condition. However, there were no differences between RALC and sham tDCS. Previous studies have shown that CBM *per se* is able to decrease craving (Fodor et al., 2017) and one possibility is that the add-on of RALC tDCS on the present form, does not augment the effects of CBM, because CBM already induces a floor-like effect. However, surprisingly, the combination of LARC tDCS and CBM suggests a task specific effect, which led to detrimental effects on chocolate craving, similar to the one showed by To et al. (2018).

Nonetheless, there are alternative explanations. Neuroimaging data suggests that in the approach avoidance conflict, there is increased BOLD activation over bilateral anterior cingulate cortex (ACC), anterior insula, caudate, and right dorsolateral prefrontal cortex (Aupperle et al., 2015). Moreover, increased right dorsolateral prefrontal cortex activation was associated with decreased approach behavior. This may explain the results of this study, as placing the cathode over the right DLPFC during the presence of craving-inducing cues, may have resulted in a net inhibition that facilitated approach to chocolate. In other words, the inter-hemispheric balance shifting toward the left hemisphere while performing CBM training resulted in a net inhibition effect over the right PFC that actually increased craving for chocolate. Additionally, when the anode is placed over the right PFC, then there is a net excitement that results in a decrease in craving (although not statistically different from sham). Thus suggesting the role of the right PFC in craving decrease during cue exposure, which is not surprisingly at all, as several studies have already suggested the relationship between the right PFC with reward feedback (HajiHosseini and Holroyd, 2015), or in its role of initiating motivated behavior (Ballard et al., 2011).

Taken together, it is plausible that the increase in craving induced by LARC may actually be the result of the temporal and spatial summation of several underlying cognitive processes. The cathode on the right may have decreased activity over the PFC and as such increased approach behavior (Aupperle et al., 2015). Moreover, the anode on the left may actually increase the activity of the left region, which has already been shown to be activated during cue-induced craving (Gordon, 2016). And as such, the present results are a summation of two distinct effects of dual hemisphere tDCS (i.e., increased approach behavior and cue-induced craving increases). The use of dual hemisphere tDCS needs to be carefully considered when combined with a task, because both the anode and the cathode will influence the network involved in task performance. For instance, in a study targeting proactive inhibition over the prefrontal cortex, the anode was placed over the right hemisphere, but the cathode dimension varied between 35 (i.e., bilateral) and 100 cm^2^ (i.e., unilateral). The unilateral stimulation was the only one that was able to induce a proactive inhibition effect, despite the fact that the anode was always placed in the right hemisphere (Leite et al., 2018).

Interestingly enough, in the present study only 22 out of 49 participants (44.90%) were able to correctly guess whether they were on active or sham tDCS which is different from the findings from O’Connell et al. (2012). It is important to highlight that there are several differences across studies. For instance, O’Connell et al. (2012) used a 5 s ramp up and ramp down process, whereas in the present study we used 15 s; additionally the blinding question was different: O’Connell et al. (2012) formulate their question toward the use of active tDCS, while in the present study, participants were asked which tDCS condition they believed they were submitted to. Also, O’Connell et al. (2012) did not test the effects of tDCS during task performance. Finally, Brunoni et al. (2014) showed that even though participants were able to correctly guess their tDCS and sertraline allocation above chance, this seemed related to treatment efficacy perception and not blinding failure.

The present study is not without limitations. First of all, the level of self-reported craving of participants. The overall level was not high, namely 2.70 points at baseline. Therefore, it is not possible to infer that the same results would be obtained from participants with higher self-reported levels of craving. Despite the fact that some studies have used non-clinical controls and showed significant results (Fregni et al., 2008; Lapenta et al., 2014), most of the studies probing the effects of tDCS on craving have been using participants with high levels of craving (Salling and Martinez, 2016; Yavari et al., 2016). Moreover, neuroimaging and neurophysiological studies have suggested several brain changes due to craving (Asmaro and Liotti, 2014), and thus the specific effects of combining tDCS and CBM in a population of high cravers should be tested in future studies.

Additionally, all the tDCS conditions in this study were applied during CBM training. Thus, in the present study it is not possible to infer that RALC or LARC tDCS *per se* is able decrease or increase craving for chocolate. Future studies should conduct full factorial trials to infer the effects of the combination of tDCS and CBM for chocolate craving. As the effects of tDCS are potentially cumulative, future studies should also test the combination of CBM and tDCS using multiple session designs. For instance, in a study by Nakamura-Palacios et al. (2016) with alcohol and crack users, it was suggested that the relapse prevention and craving reduction effects of repeated sessions of bilateral tDCS to the DLPFC was associated to an increased activation of the ventromedial PFC (vmPFC). The vmPFC is thought to be involved in emotional regulation and motivational processes that are dysregulated in people suffering from craving (Goldstein and Volkow, 2011). Thus, it is possible that single session of tDCS is not sufficient to induce a significant effect over the vmPFC through DLPFC modulation, and thus future studies should also explore the effects of multiple sessions of tDCS. Also, from the current study is not possible to infer how task and tDCS effects modulated brain activity, namely how inward and outward electrical currents changed brain activity and how the temporal and spatial properties of the resulting electric field during task performance occurred, and as such, neuroimaging methods should be used in future studies in order to assess the relationship between behavior and brain activity.

Finally, the use of other cognitive tasks targeting different aspects of proactive and reactive inhibition, impulsivity or craving *per se*, should be used in order to further test this brain laterality model.

Single session of RALC tDCS combined with CBM was able to reduce the preference for chocolate when comparing to fast food, but there were no differences when comparing to sham tDCS and CBM. Surprisingly, the combination of CBM with LARC actually increased implicit and explicit craving for chocolate when comparing to fast food. There were no effects of the combination of RALC or sham tDCS with CBM on chocolate preference. These findings suggest a tDCS state dependent effect, in which the anode placed over the left DLPFC may actually increase approach behavior for chocolate during cue exposure. These results are compatible with recent models of brain laterality, in which cue craving seems to be more dependent on the left hemisphere. Nonetheless, the combination of tDCS with CBM for decreasing chocolate craving should be tested in future studies, namely by increasing the number of sessions, and by testing such effects in a population of high cravers for chocolates.

## Author Contributions

SC and JL conceived and designed the study, acquired the data, analyzed, and interpreted the data. Substantial contributed in drafting the manuscript and revised it critically for important intellectual content, as well as final approval of the final version submitted. AS and ÓG conceived and designed the study, contributed in drafting the manuscript and revised it critically for important intellectual content, as well as final approval of the final version submitted. AM and AL acquired the data, analyzed and interpreted the data, and drafted the manuscript. DV conceived and designed the study, acquired the data, analyzed and interpreted the data, and drafted the manuscript.

## Conflict of Interest Statement

The authors declare that the research was conducted in the absence of any commercial or financial relationships that could be construed as a potential conflict of interest. The reviewer OL declared a past collaboration with several of the authors to the handling Editor.
