# Supplementary material for: Polarity Specific Effects of Cross-Hemispheric tDCS Coupled With Approach-Avoidance Training on Chocolate Craving
Source: Front Pharmacol. 2019 Jan 24;9:1500. doi: 10.3389/fphar.2018.01500 (PMC6353830; doi:10.3389/fphar.2018.01500)
Supplement: Supplementary file 1 [file Table_1.DOCX]

|  |  |  | Stimulation | | | | | | | |  |
| --- | --- | --- | --- | --- | --- | --- | --- | --- | --- | --- | --- |
|  | |  | Sham | |  | RA-LC | |  | LA-RC | | |
|  | |  | (*N* = 16) *Mean (SD)* | |  | (*N* = 16) *Mean (SD)* | |  | (*N* = 17) *Mean (SD)* | | |
|  |  |  | Pre | Post |  | Pre | Post |  | Pre | Post | |
| Implicit Association Task (IAT) | | | |  |  |  |  |  |  |  | |
| Chocolate vs Healthy Food |  |  | 0,1 (0,43) | -0,07 (0,6) |  | -0,01 (0,59) | 0,0 (0,48) |  | 0,04 (0,52) | -0,12 (0,46) | |
| Chocolate vs Fast Food |  |  | 0,25 (0,5) | 0,15 (0,4) |  | 0,46 (0,45) | 0,27 (0,42) |  | 0,02 (0,55) | 0,31 (0,4) | |
| Healthy Food vs Fast Food |  |  | 0,27 (0,54) | 0,34 (0,49) |  | 0,22 (0,35) | 0,07 (0,45) |  | 0,07 (0,5) | 0,21 (0,49) | |

Task performance on the multifactorial IAT
